# Approaches to Clinical Complete Response after Neoadjuvant Chemotherapy in Muscle-Invasive Bladder Cancer: Possibilities and Limitations

**DOI:** 10.3390/cancers15041323

**Published:** 2023-02-19

**Authors:** Hye Won Lee, Whi-An Kwon, La Ngoc Thu Nguyen, Do Thanh Truc Phan, Ho Kyung Seo

**Affiliations:** 1 Department of Urology, Center for Urologic Cancer, National Cancer Center, Goyang-si 10408, Republic of Korea; 2 Department of Urology, Myongji Hospital, Hanyang University College of Medicine, Goyang-si 10408, Republic of Korea; 3 Department of Urology, Cho Ray Hospital, Ho Chi Minh City 700000, Vietnam; 4 Department of Urology, Nhan Dan Gia Dinh Hospital, Ho Chi Minh City 700000, Vietnam; 5 Division of Tumor Immunology, Research Institute and Hospital of National Cancer Center, Goyang-si 10408, Republic of Korea; 6 Department of Cancer Biomedical Science, Graduate School of Cancer Science and Policy, National Cancer Center, Goyang-si 10408, Republic of Korea

**Keywords:** muscle-invasive bladder cancer, neoadjuvant chemotherapy, radical cystectomy, bladder preservation, clinical complete response

## Abstract

**Simple Summary:**

Neoadjuvant chemotherapy followed by radical cystectomy is the recommended standard of care for muscle-invasive bladder cancer patients who are eligible for cisplatin. Achieving a pathologic complete response in radical cystectomy provides an excellent long-term survival advantage. Since 40% of patients have a pathologic complete response, there is a growing interest in bladder preservation as a reasonable alternative strategy for oncologic control as well as improving the quality of life in these patients. However, one must be cautious when selecting a bladder preservation strategy instead of radical cystectomy because of an inaccurate restaging approach after neoadjuvant chemotherapy related to discrepancies between clinical complete response and pathologic complete response. Hence, we comprehensively discuss some of current clinical issues associated with using clinical complete response as a surrogate marker for bladder preservation, as well as for neoadjuvant chemotherapy response, and its limitation as a predictive marker for patient selection for bladder preservation in muscle-invasive bladder cancer patients.

**Abstract:**

In the surgical oncology field, the change from a past radical surgery to an organ preserving surgery is a big trend. In muscle-invasive bladder cancer treatment, neoadjuvant chemotherapy (NAC) followed by radical cystectomy (RC) is the standard of care for muscle-invasive bladder cancer (MIBC) patients eligible for cisplatin. There is a growing interest in bladder preserving strategies after NAC because good oncologic outcome has been reported for pathologic complete response (pCR) patients after NAC, and many studies have continued to discuss whether bladder preservation treatment is possible for these patients. However, in actual clinical practice, decision-making should be determined according to clinical staging and there is a gap that cannot be ignored between clinical complete response (cCR) and pCR. Currently, there is a lack in a uniform approach to post-NAC restaging of MIBC and a standardized cCR definition. In this review, we clarify the gap between cCR and pCR at the current situation and focus on emerging strategies in bladder preservation in selected patients with MIBC who achieve cCR following NAC.

## 1. Introduction

Globally, bladder cancer (BC) confers a significant disease burden due to the potential morbidity and mortality associated with high care costs and lower health-related quality of life (QOL) [1,2]. BC is ranked the 6th most common cancer in men, the 17th in women, and the 10th most frequent cancer in both sexes worldwide, with an estimated 573,278 new cases and 212,536 deaths in 2020 [1,2]. It is approximately four times more common among men than among women, with an age-standardized rate of BC incidence of 9.5 per 100,000 among men and 2.4 per 100,000 among women [1,2].

Approximately 70% of the patients with BC present with disease confined to the mucosa (stage Ta, carcinoma in situ [CIS]) or submucosa (stage T1, non-muscle-invasive BC [NMIBC]), and 25% of the patients present with a muscle-invasive BC (MIBC, stage T2–T4a) [3,4,5,6,7,8]. The standard therapeutic strategy for clinically non-metastatic MIBC is radical cystectomy (RC) with bilateral pelvic lymph node dissection (PLND) [3,4,5,6,7,8]. MIBC exhibits an unfavorable prognosis despite RC, with a 5-year overall survival (OS) rate of about 50% [2,9,10,11,12,13].

The high propensity for distant metastases and the underlying biological high susceptibility to cytotoxic chemotherapeutics underscore the importance of neoadjuvant chemotherapy (NAC) in MIBC before RC [3,4,5,6,7,8,14]. There are several theoretical advantages of administering NAC: (1) Facilitates early delivery of systemic therapy when the burden of micro-metastatic disease is low; (2) Response to NAC potentially reflects in vivo chemo-sensitivity in determining patients who do not respond to NAC at an early period or re-challenge cisplatin-based combination chemotherapy in case of recurrence in the future; (3) Better chemo-tolerability and patient compliance at the pre-RC stage, compared to adjuvant administration in the pos-RC setting [5,6,7,8,14,15,16,17]. In addition, NAC does not increase the risk of surgical morbidity [18,19]. Comparisons of RC alone with RC after NAC showed no significant differences in surgical safety, in terms of complications and hospitalization, regardless of the approach [18,19,20,21,22,23,24].

The current international guidelines recommend cisplatin-based NAC followed by RC for clinically localized MIBC based on the data from randomized controlled trials (RCTs) and meta-analysis [3,4,5,6,7,8,18,25,26,27,28,29,30,31,32,33,34,35,36,37,38,39]. In a recent meta-analysis of 17 studies that compared the efficacy of NAC + RC versus RC alone, NAC + RC significantly improved the OS (hazard ratio [HR], 0.82) [32]. Specifically, the addition of NAC conferred an absolute OS benefit of about 5–10%, with a 16–33% relative reduction in the risk of death compared to RC alone [18,19,28,29,30,32,33,34,35,36,37,40,41]. Based on this evidence, NAC is suggested to be used in cisplatin-eligible patients with MIBC and is therefore proposed as a quality indicator in RC [3,4,5,6,7,8]. Although NAC improves pathological downstaging and survival outcomes, only 30–40% of the patients experience major responses, defined as the absence of MIBC and lymph node metastasis (LNM) (<ypT2 and ypN0) in RC specimens [42]. This absence of a downstaging effect may be related to progression under systemic therapy. However, in a case-control matching study, the intermediate-term OS showed no difference between patients with residual/progressive disease post-NAC in comparison with residual/progressive disease without NAC (*p* = 0.94) [43], indicating that NAC does not seem to impair the prognosis of patients with PD after RC, even in patients who are resistant to NAC.

Although the Southwest Oncology Group (SWOG) reported a clinically successful outcome of neoadjuvant methotrexate, vinblastine, doxorubicin, and cisplatin (MVAC) for locally advanced MIBC in 2003 [18], presently, the most common cisplatin-based NAC regimens are gemcitabine and cisplatin (GC) or dose-dense MVAC (dd-MVAC), which show low morbidity, low toxicity, and good oncologic outcomes [3,4,5,6,7,8,18,25,26,27,28,29,30,37,40,44,45,46,47,48]. The shortened 2-week dd-MVAC regimen with granulocyte colony-stimulating factor (G-CSF) support was developed to improve treatment benefit of the 4-week conventional MVAC regimen by attempting to reduce significant toxicity associated with treatment interruption, delays, and early termination [49,50]. NAC with dd-MVAC has a safer profile and similar pathologic complete response (pCR) rates compared with standard-dose MVAC [51,52]. In a recent meta-analysis, dd-MVAC was superior to GC with regards to pCR defined as no evidence of residual tumor in RC and PLND specimens (ypT0N0) (35.2% vs. 25.1%; *p* = 0.006) and OS (HR, 2.16; *p* = 0.0004), suggesting that dd-MVAC is more effective than GC in patients with MIBC [27]. Contrastingly, another meta-analysis reported no significant difference in pCR, pathological partial response, and OS between GC and MVAC due to the similar curative effect and parallel long survival outcomes [25]. Therefore, the selection of GC or dd-MVAC in the clinic should be guided by further investigations using large-scale RCTs and long-term follow-up studies in the future.

There is also a lack of evidence on the optimal number of NAC cycles to be administered in MIBC. Although the NCCN Clinical Practice Guidelines in Oncology (NCCN Guidelines) for MIBC recommend four cycles of GC, three cycles are also commonly administered in clinical practice [3,4,5,6,7,8,37,44]. A retrospective study by Ferro et al. reported that three cycles of GC may be equally effective to four cycles in the NAC setting regarding pCR, pathological downstaging, and cancer-specific survival (CSS), with less long-term toxicity [44]. Similarly, there is controversy regarding the optimal number of cycles of dd-MVAC in the NAC setting [3,4,5,6,7,8,26,27,45,46,47,48]. Four retrospective studies reported the rates of pCR and pathological downstaging to be 21.0–41.3% and 37.5–69.0%, respectively, after three to four cycles of dd-MVAC, whereas, a recent RCT reported that the rates of pCR and pathological downstaging were 42.2% and 63.3%, respectively, after a six-cycle course [48]. Therefore, the ideal number of cycles for NAC warrant more rigorous prospective studies.

RC constitutes the removal of the bladder and surrounding tissues, PLND, and urinary diversion [5,6,7,8]. Therefore, the surgical duration is long with a need for extended hospital stay [53,54]. Furthermore, the rates of short-term complications (27–32%), long-term complications (64%), and mortality (1.5–5%) after RC are high, which lead to an increased interest in bladder-preserving strategies such as trimodal therapy (TMT) and partial cystectomy (PC) with NAC [5,6,7,8,37,55,56,57,58,59,60,61,62,63,64,65]. A favorable pathological response to NAC is defined as achieving ≤ypT1, ypN0, and negative surgical margins [18,33,66,67,68]. Downstaging to ≤ypT1 is associated with excellent long-term survival, while the persistent MIBC in the specimen (ypT2–4) indicates poor outcomes [67,68]. Several studies have demonstrated that achieving a pCR after NAC (ypT0N0), defined as lack of residual disease at the primary tumor site and pelvic nodes in the cystectomy specimen, is an independent factor related to excellent long-term survival, and the 5-year OS of patients with pCR is 80% [18,33,66]. Therefore, up to 40% of the patients achieving pCR following NAC may be considered for a bladder preservation strategy [33]. However, pCR after NAC can only be confirmed through RC [5,6,7,8,33].

On the other hand, a clinical complete response (cCR) after NAC is defined as absent residual tumor on cystoscope, transurethral resection (TUR), urine cytology, and cross-sectional imaging [4,5,6,7,8,29,33,37,69,70]. If the prognostic value of cCR is reliably validated as a promising alternative endpoint, accurate determination of a cCR for NAC before RC could induce a paradigm shift in bladder-preserving approaches for the management of MIBC [33,71]. However, the current lack of standardized tools for restaging and lack of a standardized definition of cCR limits its widespread clinical utility and hinders the active investigations into multidisciplinary protocols for bladder preservation. Therefore, the clinical validity of cCR as a prognostic indicator for long-term survival after NAC in MIBC requires a step-by-step and robust development to carefully establish its performance characteristics. In this review, we clarified the gap between cCR and pCR at the current situation and focuses on emerging strategies in bladder preservation in selected patients with MIBC who achieve cCR following NAC.

## 2. Morbidity and Mortality Associated with RC with PLND and Urinary Diversion: The Motivation for Bladder Preservation in cCR after NAC

The added benefit of RC with PLND in clinical complete responders is a subject of debate [59,69]. If cCR is achieved after NAC, there may be ‘pros and cons’ with the RC progress. The major drawback is that RC is associated with significant morbidity that has a profound impact on QOL. The major motivation for bladder preservation in clinical complete responders after NAC is the need to avoid complications associated with RC and PLND and urinary diversion [65,69,72,73,74,75,76,77,78]. Recent studies have demonstrated that the overall major complications (Clavien grade 3 or higher) after 30 and 90 days ranged between 25% and 30%, while the 30- and 90-day mortality rates were approximately 2% and 4%, respectively [74]. The frequency of complications increase to 94% with longer follow-up durations in the patients surviving >15 years [75]. Moreover, MIBC is a disease of the older people, a patient population that is more prone to having comorbidities that could potentially increase the rates of the postoperative complications or even might impact their eligibility to undergo RC [65,76,77,78]. Even for the patients who are eligible for surgery, RC deferment is relatively common in clinical practice due to concerns about body image that can impact the QOL of the patients [58,65,79].

## 3. Discordance between cCR and pCR: Rationale for RC and PLND in Clinical Complete Responders to NAC

Those in favor of RC believe that, first, curative-intent RC and PLND offers excellent locoregional control for patients with clinically localized or locally advanced MIBC by completely removing the affected bladder, LNs, and adjacent organ, thereby preventing the need for further treatment. Second, pCR confirmation is the best approach to avoid uncertainties about surveillance after cCR because excellent long-term survival can be achieved in the case of pT0N0. Pathologic stage at RC has a stronger association with clinical outcomes after RC [80,81]. Nearly half of MIBC patients undergoing RC have a pathologic stage discordant with their clinical stage [80,82,83], therefore, the pathology evaluation through RC and PLND has an critical impact on the outcome of patients with MIBC after RC [81]. For example, in an analysis of 16,953 patients with MIBC without distant metastases treated with RC from 1998 to 2009, a clinical–pathologic stage discrepancy rate of 47.8% (41.9% upstaging and 5.9% downstaging) was identified [82]. Shariat et al. also demonstrated in a large, contemporary, consecutive series of patients who were treated with RC and PLND that pathologic upstaging occurred in 42% of patients, and especially, 36% of patients with organ-confined clinical stage had non-organ-confined pathologic stage (pT3N0 or pTanyN-positive) [80]. This high level of discrepancy between clinical stage and pathologic stage due to inaccuracy in current pre-RC clinical staging even with the use of modern imagining techniques should be considered when selecting patients for bladder-sparing approaches. Therefore, despite a high risk of perioperative morbidity and mortality, the RC with PLND following NAC remains the gold standard option in MIBC after NAC for complete surgical resection, accurate pathologic assessment of the primary tumor and regional LNs, appropriate prognostication, and the planning of optimal adjuvant strategies. More importantly, concomitant LND after NAC offers the most reliable pathological staging for detecting LNM and helps in identifying the high-risk patients who may benefit from adjuvant treatments [69,81].With regard to the available evidence, standard PLND should be the absolute minimum approach to be performed during RC, but the therapeutic value of extended LND remains controversial [3,4,5,6,7,8,15,37,84,85,86,87].

Optimizing and standardizing cCR after NAC is crucial for the application of bladder-preserving techniques, especially in the case of MIBC [5,6,7,8,33,69,71]. In particular, the reliability of cCR as a potential surrogate marker for response evaluation of NAC relies on correlating cCR with pCR (ypT0N0) based on the final RC specimens [33,71]. The false downstaging rate reflects the continuing clinical dilemma of the inability to accurately restage the tumor status after NAC without RC. For instance, several studies have shown an insufficient degree of concordance between clinical and pathological staging of MIBC, unraveling the inadequacy of the current methods of clinical staging of patients post-NAC [14,80,88,89,90,91]. Reese et al. evaluated the utility of an extensive restaging approach following NAC before RC in 62 patients with MIBC using clinical staging examinations including cystoscopy, bimanual examinations, and abdominal–pelvic cross-sectional imaging [88]. Clinically, cCR was evident in 35% (22/62) of the patients before RC, while pCR was evident in 25% (12/62) of the patients after RC, showing a significant difference between cCR and pCR [88].

Recent studies demonstrated that the absence of residual tumor during repeated TUR of bladder tumor (TURBT) after NAC does not necessarily predict pT0 at RC [91,92,93,94]. The SWOG 0219 (S0219) phase II trial evaluated the clinical T0 (cT0) rate with three cycles of neoadjuvant paclitaxel, carboplatin, and gemcitabine as well as TURBT for patients with histologically confirmed non-metastatic MIBC (cT2–T4a) [91]. After NAC was completed, patients were to undergo another re-staging TURBT to assess the cT0 rate of this NAC regimen [91]. Patients with cT0 status after NAC could elect immediate RC or cystoscopic surveillance, and those with greater than cT0 status (residual tumor at the post-NAC TURBT) were to undergo immediate RC [91]. Of the 34 patients with cT0 status after NAC 10 underwent immediate RC, 6 of whom had persistent cancer at RC (greater than pT0 (pT2–T4)) [91]. This unacceptably high rate (60%) of persistent cancer at RC in patients presumed to have pT0 status suggests the need for definitive local treatment such as RC rather than cystoscopic surveillance, regardless of post-NAC cT0 status, in patients completing NAC. Similarly, Kukreja et al. investigated patients with cT0 at repeat TURBT who then went on to undergo RC, and found that residual tumor at RC was reported in 63.7% (101/157) of all cT0 patients at repeat TURBT (no residual disease on biopsy) [92], indicating that complete TURBT does not predict pT0 at RC. A notable fraction of patients with cT0 bladders (24.8%, 39/157) had locally advanced (≥pT3) and/or LNM [92]. Almost half of the patients (49.7%, 78/157) received NAC, but there was no difference in primary outcome for pT0 status at RC between patients with and without NAC (53.6% vs. 46.4%, *p* = 0.47) [92]. Furthermore, a retrospective study reported the fate of 63 patients with MIBC who declined definitive RC after achieving a cCR (cT0) after NAC (defined as no residual disease at first follow-up on any of abdominal and pelvic CT scan, urine cytology, and cystoscopy with TUR biopsy) [94]. Overall, 40 of the 63 patients (64%) had recurrent tumors in the bladder, which was muscle-invasive in 24 (38%) and noninvasive in 16 (25%) [94].

In a recent retrospective study to evaluate the ability of restaging TUR following NAC and prior to RC as a clinical restaging tool after NAC through direct comparison of post-NAC and RC pathology, 46% (53/114) of the patients were restaged and had rcT0 (no residual disease in restaging TUR); however, of these, only 47% (25/53) had rpCR (ypT0 on final RC pathology) [93], indicating the abundance of many variations in the cCR, even when evaluated using TUR (Table 1). Furthermore, 32% (37/114) of the corresponding patients were falsely down-staged (<rT2 to ≥ypT2) and 23% (12/53) of rcT0 were up-staged more than pT2 [93]. This level of inaccuracy is intolerable for guiding management decisions, especially the choice to forego standard consolidative therapy. These findings support prospective trials such as the RETAIN BLADDER (NCT02710734) [95], Alliance A031701 (NCT03609216) [96], and HCRN GU16-257 (NCT03558087) [97] that emphasize the importance of evaluating the role of post-NAC restaging TUR and risk-adapted approach to treatment of MIBC (Table 2).

The 5-year recurrence-free survival (RFS) and OS for patients with pN+ (21–42% and 25–38%, respectively) are significantly worse than those with pN0 (56–78% and 49–69%, respectively) [11,86,98,99,100,101,102]. The previously reported frequency of LNM in the RC specimen was 16–35% [86]. NAC before RC is associated with a lower incidence of pN+ than that of RC alone [3,4,5,6,7,8,18,25,26,27,28,29,30,31,32,33,34,35,36,37,38,39,86,103], and the persistent LNM in RC specimens despite NAC associated with poor prognosis suggests a diagnostic role of LND [103]. Reliance on cCR in determining surgical omission assumes that the nodular micro-metastases share similar chemo-sensitivities to the primary bladder tumor and that PLND could be omitted [104]. However, this may not be the case because of the intra- and inter-tumoral genomic heterogeneity. Furthermore, the ypT0N+ pathology has prompted concerns about post-NAC bladder preservation without PLND [69,104,105]. In a recent study that investigated the prevalence of occult LNM in patients with or without NAC, occult LNM was evident in 4.3% of the patients with cT2-4aN0M0 MIBC with complete downstaging of the primary tumor following RC plus PLND [105]. Nassiri et al. also recently highlighted the discrepancy between the primary tumor and nodal stages in a review of the patients with non-metastatic MIBC who underwent NAC followed by RC and PLND within the National Cancer Database [69] and found occult LNM in 4.9% of the patients with a cCR after NAC and 5.4% of the patients who were down-staged to NMIBC [69].

Based on these findings, PLND should be considered in the patients with a cCR after NAC to provide valuable prognostic information. However, the evidence is limited in terms of the adequate extent of LND and the therapeutic role of LND in RC after NAC [15,84,85,86,87]. For instance, von Landenberg et al. reported no significant association between LN yields and survival outcomes in patients undergoing NAC [87], suggesting that an adequate LND (defined as ≥10 LNs removed) could not significantly improve survival in patients treated with NAC because therapeutic roles of LND via the eradication of micro-metastasis to LNs may be already achieved by NAC [15,84,85,86,87]. The optimal LND template as well as the diagnostic and therapeutic role of LND for post-NAC RC should be further investigated.

## 4. A Review of Various Strategies for Post-NAC Clinical Assessment

Common approaches for post-NAC restaging include cross-sectional imaging (computed tomography [CT]/magnetic resonance imaging [MRI]), cystoscopic examination with or without TUR or biopsy, urine cytology, bimanual examination, or a combination of these procedures [4,5,6,7,8,29,33,37,69,70]. A bone scan can be performed if there are bone-related symptoms or elevated levels of alkaline phosphatase [37]. Flourine-18 fluorodeoxyglucose positron emission tomography/CT (^18^F-FDG PET/CT) might be beneficial in some cases, but is not recommended for the routine staging of MIBC [4,5,6,7,8,29,33,37,70].

### 4.1. Cystoscopy and TUR in Post-NAC Restaging

The gold standard in diagnosis and follow-up of BC is cystoscopy; however, limitations include the possibility of missing flat and sessile lesions and the absence of the assessment of nodal and extravesical involvement [3,4,5,6,7,8,106,107]. A prospective single-arm study evaluating the reliability of the Systematic Endoscopic Evaluation (SEE) in predicting pCR (NCT02968732) developed a standardized scoring system and bladder map diagram for objective quantification of cystoscopic findings [108]. Biopsies of the visible and/or prior tumor sites along with two random sampling of the normal-looking mucosa were performed in all patients [108]. Among 50.8% (31/61) patients with no visual or biopsy-based tumor on SEE, 51.6% (16/31) patients harbored a residual disease (>pT0), including 25.8% (8/31) with MIBC (≥pT2) upon RC, reporting the negative predictive value (NPV) of SEE for predicting a pCR (48.4%) below their pre-specified hypothesis [108]. In another prospective study at four Swedish cystectomy centers including 46 patients with MIBC who underwent cystoscopy after NAC, immediately before RC [109], the correlation between visual assessments using cystoscopy and the pathologic findings in RC specimens was analyzed. Unfortunately, the cystoscopy falsely predicted ypT0 in 50% (14/28) among the 28 patients, with no signs of residual tumors during cystoscopy [109]. The diagnostic accuracy of post-NAC clinical restaging based on cystoscopy was unsatisfactory, with a sensitivity of 64%, a specificity of 82%, a positive predictive value (PPV) of 89%, and NPV of 50% [109], implying that cystoscopy after NAC cannot safely determine complete responders who were appropriate candidates for bladder preservation. Recently, the enhanced cystoscopic techniques including fluorescence and narrow-band imaging present potential adjunctive procedures with regards to restaging following NAC by improving the visualization of the residual tumor [3,4,5,6,7,8,33,106].

### 4.2. CT/MRI in Post-NAC Restaging

Conventional imaging modalities like CT and MRI are often ineffective in response assessment due to difficulties in identifying a viable tumor deposit in bladder and small tumors in normal sized LNs [110]. A real-world retrospective multicenter study of 242 patients with MIBC (cT2N0M0-cT4aN0M0) investigated the discrepancies between nodal stage assessed by CT and pN state [111]. In total, 28 of 133 (21%) patients, who were considered having no nodal progression in CT, presented with nodal progression at the post-RC histopathological staging, and CT was able to detect only 3.4% (1/29) of the patients with nodal progression [111]. Overall, the sensitivity and specificity of CT in predicting pN(+) status were 17% and 78%, respectively [111].

Recently, diffusion-weighted (DW) MRI was used to predict the response to NAC, owing to its capability of providing functional information on tumors in terms of cellularity and disorganization of the tumor tissue. Ahmed et al. evaluated the diagnostic performance of dynamic contrast enhanced-(DCE) and DW-MRI in the assessment of post-NAC cCR [112] and prospectively enrolled 90 patients with MIBC post-NAC. The sensitivity and specificity for predicting pCR using the apparent diffusion coefficient (ADC) value and wash-out rate cut-off values were 95.4% vs. 97.7% for reader 1, and 96% vs. 97% for reader 2, respectively, suggesting that DW-MRI is a potential indicator for predicting pCR to NAC in MIBC [112].

The Vesical Imaging-Reporting and Data System (VI-RADS), based on multiparametric MRI (mpMRI), is a novel standardized approach for preoperative MIBC staging and assessment of therapeutic effects of NAC [113,114,115,116,117]. However, clinical utility and diagnostic accuracy of VI-RADS score as a predictor of pCR should be further verified for selecting the optimal candidates for bladder preservation. A recent study investigated the clinical feasibility of a newly defined categorical scoring, the NAC VI-RADS (NacVI-RADS), to determine treatment response to NAC based on radiologic assessment of response (RaR), and categorized the NacVI-RADS scoring using the prior VI-RADS score, presence of residual tumor, tumor size, and infiltration of the muscularis propria [114]. In 10 patients with non-metastatic MIBC who underwent mpMRI after NAC before RC, the NacVI-RADS categories could match all the final RC pathologic findings, for patients defined as both pCR and partial or minimal responses [114], suggesting its clinical applicability for therapeutic decisions through bladder preservation or RC.

### 4.3. ^18^F-FDG PET/CT in Post-NAC Restaging

Integrated F-FDG PET/CT is an anatomo-metabolic imaging modality that has the potential to significantly reduce the false-positives of PET and CT, which are performed separately [106,110,118,119,120,121]. Currently, ^18^F-FDG PET/CT is not sufficiently accurate for the evaluation of a primary tumor (T-staging) because ^18^F-FDG excreted in the urine and accumulated in the bladder masks the uptake of any bladder wall lesions, but is used for detecting metastasis (NM-staging) in the primary staging of MIBC and for recurrence after RC [3,4,5,6,7,8,106,110,118,119,120,121]. Several methods have also been suggested to reduce the urine FDG activity, such as bladder irrigation, catheterization, FDG wash-out, dual phase imaging, early PET images, late PET images after voiding, oral hydration, and forced diuresis [106,110,118,119,120,121]. A recent study to evaluate the potential value of ^18^F-FDG PET/CT for predicting the NAC response in patients who received ^18^F-FDG PET/CT before and after NAC followed by RC reported the detection of a partial response and complete response with a sensitivity of 70% and 67% and a specificity of 71% and 75%, respectively [122]. However, the observed NPV for pCR was still low, especially in LNs, indicating the presence of residual tumor in the case of a negative LN from ^18^F-FDG PET/CT after NAC. Similarly, Kollberg et al. assessed two ^18^F-FDG PET/CT examinations at baseline and after three cycles of platinum-based NAC in 50 patients with oligometastatic MIBC [123]. ^18^F-FDG PET/CT prediction of chemotherapy response in nodal status was accurate in 37 of 43 patients with pN+, demonstrating 86% sensitivity in the LN status prediction after NAC [123]. The role of ^18^F-FDG PET/CT for response assessment after NAC needs to be further evaluated in large-scale clinical trials in MIBC.

### 4.4. Minimal Residual Disease Monitoring Using Liquid Biopsy in MIBC

The main purpose of NAC is to control micro-metastasis before RC. Liquid biopsies are minimally invasive, measured in blood or urine, rendering them appealing to guide the perioperative management of patients with MIBC [124]. Micro-metastases can be confirmed by ‘liquid biopsies,’ using circulating tumor cells (CTCs), cell-free DNA (cfDNA), and plasma-circulating tumor DNA (ctDNA), which are a reflection of the tumor burden [14,124]. The combined approach of liquid biopsy and improved imaging modalities may hold a promise in selecting the patients with MIBC for bladder preservation. However, prospective studies, rather than proof-of-concept studies, are urgently needed to investigate the actual clinical applicability for the treatment decision-making.

In particular, ctDNA—which is the mutated cfDNA originating from cancer cells—can be used to select the patients with no minimal residual disease for bladder preservation following NAC. To identify ctDNA, tumor-specific genomic alterations are determined by referencing the germline sequence data obtained from the same patient [125]. However, ctDNA sometimes comprises <1% of the total cfDNA, rendering its detection challenging [124,125,126,127,128,129]. As a DNA-based biomarker, ctDNA offers stability in the expression levels for monitoring the disease status before and during NAC in patients with MIBC [14,124,125,126,127,128,129,130,131]. Notably, ctDNA, which is detectable in 14% of the patients with localized MIBC, has been suggested to be a promising tool to assess the therapeutic response to NAC and to detect/quantify systemic minimal residual disease (MRD), particularly in the post-NAC setting [14,124,125,126,127,128,129,130,131,132,133]. Christensen et al. unraveled the promising role of ctDNA dynamics during NAC as a predictor of response and clinical outcomes in 68 patients with MIBC by analyzing longitudinal ctDNA samples before and after NAC and after RC [131]. They performed whole exome sequencing of the tumor tissues to identify the patient-specific somatic variants and then probed the ctDNA for the presence of 16 patient-specific somatic variants by ultra-deep sequencing (multiplex polymerase chain reaction next-generation sequencing) of the plasma cfDNA [131]. Positivity for ctDNA after NAC and before RC was significantly associated with shorter RFS and OS [131]. Patients with detectable ctDNA after NAC and prior to RC were much more likely to experience recurrence following RC (75% vs. 11% at 12 months) [131]. More importantly, all the ctDNA-positive patients had residual tumor and/or LNM at RC, and all the patients (36/36) with ypT0 at cystectomy were ctDNA-negative [131]. Interestingly, 9/17 (53%) patients with ctDNA clearance after NAC experienced pathological downstaging compared with none in those with persistent DNA [131]. For the patients who were ctDNA-positive before or during NAC, the ctDNA dynamics during NAC showed a superior association with the outcomes of the patients compared to the pathological downstaging [131].

More importantly, in MIBC, the close proximity of tumor to the urine can contribute to a greater accumulation of tumor derived mutant DNA (mutDNA) [134]. Patel et al. analyzed 248 liquid biopsy samples including plasma, urine cell pellet (UCP), and urine supernatant (USN) collected from 17 patients with MIBC undergoing NAC using a combination of Tagged-Amplicon Sequencing (Tam-Seq) and shallow Whole Genome Sequencing (sWGS) [130]. MutDNA presence and levels were compared between matching plasma, UCP, and USN samples in order to determine the optimum sample type for mutDNA analysis [130]. MutDNA was detected in 35.3%, 47.1%, and 52.9% of pre-NAC plasma, UCP, and USN samples, respectively, and mutDNA was detected more frequently and at higher levels in urine (both USN and UCP) (*p* ≤  0.001) [130]. Of note, persistence of mutDNA detection at the second cycle of NAC using these methods was indicative of early disease recurrence with a sensitivity of 83% and specificity of 100%, emphasizing its potential as an early biomarker for NAC response [130]. Chauhan et al. applied urine Cancer Personalized Profiling by Deep Sequencing (uCAPP-Seq), a targeted next-generation sequencing method for detecting urine tumor DNA (utDNA), to urine cfDNA samples acquired on the day of RC from 42 patients with non-metastatic bladder cancer [132]. Among 32 pathologically confirmed MIBC patients at RC, 59% (19/32) received NAC [132]. When patients were classified as those who had residual disease detected in the RC specimen (n = 16) compared to those who achieved a pCR (n = 26), median utDNA levels were 4.3% vs. 0%, respectively (*p* = 0.002) [132]. Using an optimal utDNA threshold to define MRD detection, positive utDNA MRD detection prior to RC was highly correlated with the absence of pCR with a sensitivity of 81% and specificity of 81% [132]. Moreover, utDNA MRD-positive patients exhibited significantly worse PFS (HR = 7.4, *p* = 0.02) compared to utDNA MRD-negative patients [132]. Chauhan et al. also profiled utDNA in 74 localized BC (58 MIBC and 16 treatment refractory NMIBC) patients using ultra-low-pass WGS (ULP-WGS) and uCAPP-Seq to sensitively detect MRD in urine and accurately predict survival after NAC and curative-intent RC [133]. A random forest model incorporating variant allele frequency, inferred tumor mutational burden, and copy number-derived tumor fraction levels in urine cfDNA was 87% sensitive for predicting MRD in reference to gold-standard RC pathology [133]. These multi-modal urine cfDNA methods to sensitively detect MRD and predict pCR in MIBC after NAC. Therefore, utDNA MRD detection status after NAC but before RC for MIBC has the potential to facilitate more personalized treatment interventions for MIBC in the future, enabling clinicians to select patients for curative-intent bladder-sparing treatments.

In summary, ctDNA and utDNA monitoring in patients with MIBC who undergo NAC and RC may be useful in the early risk stratification, prediction of treatment response, and early detection of metastatic relapse. More RCTs are needed to determine the clinical impact of liquid biopsy-stratified therapeutic planning in patients with MIBC treated with NAC.

## 5. Current Strategies of Bladder Preservation in the Case of cCR

There are different bladder-preserving protocols (BPPs) such as unimodal chemotherapy or radiotherapy, maximal TURBT or PC, and multimodal strategy. There are pros and cons for performing PC in case of cCR after NAC, and those in favor believe the following: (1) the morbidity of PC is lower than that of RC, and urinary tract diversion is not required, and (2) the pathological confirmation is possible. The ideal candidates are patients with a solitary lesion < 3–4 cm, where excision of 2 cm margins is feasible (such as the bladder dome), and there is no concomitant CIS, no need for ureteral re-implantation, and no hyper-contractility of the bladder [37,58,59,60,61,62,63,64,65,135]. In highly selected patients, PC may provide reasonable oncological outcomes, with the added advantage of accurate staging by LND and full-thickness resection with adequate evaluation of surgical margins compared to TURBT alone [37,58,59,60,61,62,63,64,65,136]. However, less than 5% of the patients with MIBC would meet the criteria for PC [137], and there are concerns about its oncological outcome compared to that of RC. Bazzi et al. retrospectively reviewed 36 patients with MIBC (all with a solitary tumor < 5 cm) who underwent PC after NAC at the Memorial Sloan Kettering Cancer Center between 1995 and 2013 [136]. The majority of patients received platinum-based NAC, with 20 (56%) receiving GC combination [136]. In all patients who underwent clinical restaging following NAC, 21 (58%) achieved rcT0, 3 (8%) had rcTis, and 4 (11%) had rcN+, whereas in patients with PC pathological findings, ypT0 was seen in 18 (50%) patients, ypTis was seen in 6 (17%), ypN+ was seen in 4 (11%), and positive surgical margin was seen in 3 (8%) patients [136]. Notably, 7 (33%) of the 21 patients who were cT0 following NAC had residual tumor in the PC specimens [136]. At last follow-up, 19 (53%) patients had recurrence, 9 had recurrence in the bladder, with 6 in the bladder only [136]. Overall, 20 (56%) patients showed no evidence of disease after median follow-up of 17 months, with 15 having had no recurrences [136]. Overall 5-year RFS, advanced RFS, and OS were 28%, 51%, and 63%, respectively [136]. After NAC, the presence of CIS was associated with worse OS, while cN+ was associated with worse RFS and OS [136], which suggests that PC following NAC provides acceptable oncological outcomes in highly selected patients with MIBC.

Multimodal (TMT or tetra-modal) strategy is the most promising and commonly used approach [37,58,59,60,61,62,63,64,65]. TMT regimens vary among trials, but generally consist of maximal TURBT followed by radiotherapy with concurrent radio-sensitizing chemotherapy, with either neoadjuvant or adjuvant combination chemotherapy as well [65,138]. An alternative bladder-preserving option that has been recently evaluated is the tetra-modal therapy, consists of a maximal TURBT followed by concurrent chemoradiation and consolidative PC with PLND [3,4,5,6,7,8,30,37,58,59,60,61,62,63,64,65,70,139]. If residual disease is present at restaging after BPPs, salvage cystectomy is recommended [5,6,7,8,37,58,59,60,61,62,63,64,65]. Therefore, multimodal BPPs require very close multidisciplinary cooperation and a high level of patient compliance. Whilst previously offered only to older or frail patients who are medically unfit for RC and those looking for an alternative to RC [140,141], the current international guidelines recommend BPPs as viable options to all suitable patients, especially [5,6,7,8,37,58,59,60,61,62,63,64,65]. The patient selection is of paramount importance for the successful oncological control in BPPs. The ideal candidates for BPPs include patients who have a unifocal tumor < 5 cm that can be completely visibly resected, absence of CIS, no evidence of palpable mass at bimanual examination, cT2–T3a, no diffuse bladder wall enhancement or extravesical or nodal disease in the cross-sectional imaging, no hydronephrosis, and a good bladder function and capacity [37,58]. However, it is still controversial whether a patient with cCR after NAC can be a good candidate for BPPs in MIBC.

The characteristics linked to worse outcomes with TMT include multifocal disease, diffuse CIS, locally advanced disease (cT3/T4), hydronephrosis, and incomplete TURBT or inability to perform maximal TURBT [58]. Current clinical evidence strongly support TMT as an acceptable BPP in terms of oncological outcomes, long-term survival rates, and QOL in appropriately selected patients with MIBC, despite conflicting data from the literature and the lack of large scale multi-center RCTs comparing TMT and RC [3,4,5,6,7,8,30,37,55,56,57,58,59,60,61,62,63,64,65,70,93,139,142,143,144]. For example, Mak et al. performed a Radiation Therapy Oncology Group (RTOG) pooled analysis (five phase II studies (RTOG 8802, 9506, 9706, 9906, and 0233) and one phase III study (RTOG 8903)) of long-term outcomes of selective bladder-preserving combined-modality therapy (CMT) in 468 patients with MIBC (cT2 61%, cT3 35%, cT4a 4%) [143]. Complete response to CMT was documented in 69% of patients [143]. They demonstrated that this treatment approach results in low rates of muscle-invasive local failure (10-year, 14%) and high long-term DSS (5- and 10-year DSS, 71% and 65%, respectively) and OS (5- and 10-year OS, 57% and 36%, respectively), with 80% of patients retaining an intact bladder at 5 years [143]. Similarly, Giacalone et al. reported the long-term outcomes of TMT based on an updated retrospective analysis of 475 patients with cT2-T4a MIBC enrolled on RTOG protocols or treated as per RTOG protocol at the Massachusetts General Hospital between 1986 and 2013 [144]. Overall, 75% of patients achieved a CR to chemoradiation [144]. The 10-yr rates of muscle-invasive, regional LN, and distant failures were 18%, 14%, and 35%, respectively [144]. This consecutive single-institution experience of TMT demonstrated favorable long-term DSS (10-year, 59%) and OS (10-year, 39%), with bladder preservation (10-year bladder-intact DSS, 46%) possible in over 70% [144]. Furthermore, our analyses show marked improvements in DSS when evaluated over time, with 5-yr DSS approaching 85% in the modern treatment era, likely due to evolving criteria for patient selection across the range of our study period [144]. These findings demonstrate high rates of CR and bladder preservation in patients receiving TMT, confirm DSS rates similar to modern RC series for patients with similarly staged MIBC, and continue to support and establish the role for bladder-preserving TMT as an attractive alternative to RC for well-selected patients with MIBC motivated to preserve their native bladder. In addition, compared to the patients who underwent RC, TMT was associated with a higher QOL, including better social, physical, sexual, and cognitive functioning [57]. Patients who do not achieve a cCR (i.e., non-responders or partial responders) or who experience invasive recurrences after cCR following TMT are advised to undergo salvage RC [3,4,5,6,7,8,30,37,58,59,60,61,62,63,64,65,70,139]. Recent studies comparing salvage RC to primary RC demonstrate no significant differences in the rates of major complications or perioperative mortality but find a modest increase in minor complications [145,146,147]. Although the 5-year and 10-year CSS rates for patients treated with salvage RC in both settings (non-responders and for recurrent tumor) are 50–60% and 40–50%, respectively [143,144,145,148], it is critical to provide appropriate patient counseling regarding salvage RC.

Since distant metastasis after BPPs are comparable to that seen with RC [149,150,151], the rationale for using NAC prior to BPPs is also applicable. The rationale for NAC prior to BPPs is the same as for patients undergoing RC: to achieve adequate tumor debulking, eradicate micrometastases, decrease recurrence rates, and improve OS [65]. More recent retrospective reports have evaluated and confirmed both the safety and efficacy of NAC followed by concurrent chemoradiation, with CR, OS, and CSS rates ranging from 73% to 86%, 68% to 72%, and 76% to 79%, respectively [152,153,154,155]. However, there is limited evidence for the use of NAC with TMT due to many limitations of previous chemoradiation trials in MIBC assessing the survival benefit of NAC and a lack of data from large RCTs with adequate NAC. Moreover, previous prospective trials and retrospective studies evaluating NAC prior to TMT reported conflicting results [149,156,157,158]. RTOG 8903, the first phase III trial of NAC associated with BPPs using chemoradiotherapy (two cycles of neoadjuvant cisplatin, methotrexate, and vinblastine (CMV) vs. no CMV), was closed prematurely after accruing only 126 patients (71%) due to unexpectedly high rates of severe neutropenia and sepsis [149]. After a median follow-up of 60 months, there were no significant differences in pCR rate or OS between the arms [149]. The limitation of this study is that MCV is not currently a standard NAC regimen for MIBC [149]. In contrast, in a prospective cohort study by Thompson and colleagues [156], patients were selected for NAC on the basis of performance status, comorbidities, and renal function; all patients received 3–6 cycles of platinum-based NAC followed by concurrent chemoradiation with gemcitabine as the radiosensitizer [156]. Of the 78 patients, 38 received NAC and 40 did not [156]. There was no additional toxicity seen in the NAC arm, treatment completion rates were acceptable, and DFS and OS were similar [156]. Complexity and contradiction in these studies do not support the routine addition of NAC to curative-intent BPPs for MIBC, and optimizing the NAC sequencing and regimens for bladder-preserving approaches to MIBC should continue to be further studied under RCTs in properly selected patients.

On the other hand, the goal of tetra-modal therapy is to overcome the limitations of TMT which include the subclinical residual disease in the original MIBC site and the lack of PLND [65]. Although the role of PLND has not been established for TMT, remarkable data were recently published from a Japanese prospective study on the tetra-modal therapy in which the patients with solitary MIBC with no involvement of bladder neck or trigone and no residual disease (or minimal NMIBC disease) after chemoradiation were offered consolidative PC with PLND for bladder preservation [159]. The study enrolled 154 patients with MIBC using a tetra-modal BPP that consists of debulking TURBT followed by 40 Gy irradiation with concomitant cisplatin [159]. After concurrent chemoradiation, 81% (125/154) of the patients had a complete remission of MIBC and 69% (107/154) of the patients completed a planned tetra-modal protocol [159]. Of these patients, 90% (96/107) achieved pT0 and 2% had LNM on pathology, while 18% of the patients experienced local recurrence, including 4% with MIBC [159]. These patients showed a favorable oncological outcome with the 5-year RFS, CSS, and OS rates of 97%, 93%, and 91%, respectively, while the functional and health-related QOL outcomes remained unaltered [159], which highlights the potential role of the tetra-modal bladder-preservation approach in patients with MIBC [159]. However, this approach is still considered experimental and further larger scale trials are required to investigate and evaluate the reproducibility of tetra-modal BPP.

## 6. Real World Clinical Practice and Future Perspectives

The use of NAC for patients with MIBC has increased over the past 15 years based on landmark RCT data supporting the OS benefits [18]. However, clinical trials might not accurately reflect the real-world data [160,161]. The patients in clinical trials are often healthier, which could affect the type and quality of chemotherapy they receive. The disease status of the patients in a trial may not always reflect what is observed in the real world. The patients might show more symptoms including hydronephrosis and gross hematuria. The timing of interventions and assessments is often standardized in trials, but often could not be generalized in real clinical practice. Several factors may affect the timing of treatment, interventions, and assessments, such as insurance, medical accessibility, and physician schedules, which often leads to delays in receiving the actual interventions of NAC to RC. While the RCT data demonstrate a clinically relevant OS benefit for NAC, retrospective real-world data are less clear [6]. In a large multicenter real-world study including 935 patients in 19 centers, most patients (602, 64.4%) received GC, followed by MVAC (183, 19.6%) and other regimens (144, 15.4%); the rates of ypT0N0 and ≤ypT1N0 were 22.7% and 40.8%, respectively [42]. The adjusted pCR rate (pT0N0) (ypT0N0 disease at RC) for patients receiving GC was 24.5% compared with 25.1% for MVAC (*p* = 0.86) and showed no statistically significant difference between GC and MVAC in ypT0N0 in the multivariable analysis (OR: 0.89, *p* = 0.6) [42]. Remarkably, the pCR rate and the median OS were 23% and 5.8 years versus 38% and 6.4 years in the NAC arm of the pivotal trial (SWOG 8710) [18]. However, these data were collected retrospectively at 19 centers on patients with non-metastatic MIBC who received at least three cycles of NAC, followed by RC, between 2000 and 2013. An important limitation is the use of standard MVAC regimen that is no longer preferred NAC for MIBC. Recently, MVAC has been replaced by dd-MVAC with GF support, which has been shown to have good comparable tolerance and a higher pCR rate than standard MVAC [42,52,162]. Similarly, a recent national analysis showed ypT0 rates as low as 13% [163], highlighting the need to lower the expectations before extrapolating the clinical trial evidence to patients in the real clinical practice.

Although NAC can reduce the risk of distant spread in MIBC patients, 5–10% of patients remain unresponsive, resulting in potentially fatal RC delays and treatment toxicity [2,3,4,5,6,7,8,9,10,11,12,13]. It is crucial to stratify MIBC patients by NAC efficacy for distinguishing between chemo-sensitive patients who can achieve cCR following NAC opting for BPP and high-risk MIBC patients who can benefit from RC following NAC, likely owing to the presence of residual disease and micro-metastasis. Currently, patients with a pCR after NAC who are eligible for RC cannot be accurately identified. The unsolved issue before embarking on BPPs after NAC is that the current imaging modalities lack accuracy in assessing micro-metastasis or rT staging after NAC. To identify the patients who are likely to benefit from the bladder preservation strategy after NAC, more accurate restaging tools are required, including novel endoscopic techniques, advanced imaging, histopathologic/genomic/molecular prognostic or predictive biomarkers, and liquid biopsies (blood/urine biomarkers). For example, in recent years, biomarkers to help predict response to cisplatin-based NAC have been studied most extensively, mainly focusing on ERCC excision repair 2 (ERCC2) [164]. The antitumor mechanism of cisplatin as an alkylating agent is based on the induction of DNA damage either as single-strand breaks, double-strand breaks, or interstrand-crosslinks, ultimately interfering with DNA replication and inducing cell death [165,166]. Cisplatin-induced DNA damage can be repaired by the nucleotide excision repair (NER) and homologous recombination pathways [165,166]. As the ERCC excision repair 2 (ERCC2) repair single-strand breaks via the NER pathway, which repairs single-strand breaks [167], the ERCC2 mutations found mostly in the helicase domain confer sensitivity to cisplatin due to loss of NER capacity [164,168]. Van Allen et al. prospectively applied whole exome sequencing to pre-NAC tumor and germline DNA in 50 patients who received cisplatin-based NAC followed by RC (responder (≤ypT1), n = 25; non-responders (≥pT2), n = 25) [169]. Specifically, ERCC2 was the only gene significantly enriched in the responder cohort (36%) (vs. 0% in the non-responder cohort) [169]. These findings were confirmed in an independent validation cohort where 8/20 responders (40%) compared to 2/28 non-responders (7%) had an ERCC2 mutation [170]. Similarly, in an independent retrospective cohort of 165 patients with MIBC who subsequently underwent NAC and RC [171], somatic deleterious mutations in ERCC2 detected in pretreatment TUR material were found in 9 of 68 (13%) evaluable responders (ypT0/Tis/Ta/T1N0 disease after RC) and 2 of 95 (2%) evaluable non-responders (*p* = 0.009) [171]. In summary, current evidence confirms that the analysis of deleterious mutations in ERCC2 can provide predictive information in MIBC patients treated with cisplatin-based NAC. They should be further validated through well-designed RCTs.

## 7. Conclusions

Patients with MIBC who achieve pCR after NAC have favorable CSS and OS. With the increasing number of patients who receive NAC and 40% of patients with pCR in RC, there is a growing interest in bladder preservation for these patients. However, in actual clinical practice, decision-making should be determined according to clinical staging and there is a gap that cannot be ignored between cCR and pCR. There is still insufficient evidence to support a change in the NAC paradigm based on cCR. Although the options for definite local treatment are evolving, BPPs in patients who achieve a cCR after NAC should be considered only in highly selected patients because of the risks of inaccurate clinical staging and possibility of micro-metastases, and close monitoring and salvage cystectomy in patients with recurrence should be performed. Prospective clinical trials investigating bladder-preserving approaches after cisplatin-based NAC should incorporate uniform restaging procedures and validated molecular diagnostics for better evaluation of the response to NAC. Clinical trials on this are currently underway. A change in the paradigm of MIBC treatment may be expected if a bladder preservation strategy shows favorable outcomes using clinical, radiological, and molecular parameters.

## Figures and Tables

**Table 1:** Distribution of clinical and pathological stages in patients undergoing post-NAC restaging TUR (n = 114) [93]. NAC, neoadjuvant therapy; RC, radical cystectomy; TUR, transurethral resection.

	Final RC Specimen Pathology Stage Following NAC (ypT), N (%)							
Post-NAC TUR Pathology (rT), N (%)	ypT0	ypTa	ypTis	ypT1	ypT2	ypT3	ypT4	Total
rT0	25 (47)	1 (2)	13 (25)	2 (4)	5 (9)	4 (8)	3 (6)	53 (100)
rTa	1 (10)	2 (20)	1 (10)	1 (10)	1 (10)	2 (20)	2 (20)	10 (100)
rTis	2 (22)	0 (0)	5 (56)	1 (11)	1 (11)	0 (0)	0 (0)	9 (100)
rT1	0 (0)	0 (0)	3 (12)	3 (12)	6 (24)	7 (28)	6 (24)	25 (100)
≥rT2	0 (0)	0 (0)	0 (0)	3 (18)	1 (6)	8 (47)	5 (29)	17 (100)
Total	28	3	22	10	14	21	6	114

Note: Diagnostic performance of post-NAC restaging TUR (≥rT2) for residual MIBC at RC (≥ypT2); Specificity: 60/63 = 95%/Sensitivity: 14/51 = 27%/Negative predictive value: 60/97 = 62%/Positive predictive value: 14/17 = 82%.

**Table 2:** Ongoing clinical trials to evaluate risk-adapted approach to treatment of MIBC.

Study Name	Design	NAC Regimens	Methods/Primary Endpoint/Interim Results
RETAIN BLADDER (NCT02710734) [95]	Multicenter, prospective, phase II trial	Accelerated methotrexate, vinblastine, doxorubicin, and cisplatin	▪ 71 patients with cT2-T3N0M0 MIBC ▪ Pre-NAC TURBT specimens were sequenced for mutations in ATM, ERCC2, FANCC, or RB1 (Caris Life Sciences). Patients with at least one mutation and no clinical evidence of disease by restaging TUR and imaging post-NAC began pre-defined active surveillance (AS). ▪ Primary endpoint: Metastasis-free survival (MFS) at 2 years ▪ Interim results: Risk enabled therapy utilizing the selection of clinical and genomic factors in patients with cT2-T3 MIBC demonstrates a 50% rate of any tumor recurrence and a 11% rate of locally advanced/metastatic disease in the AS group.
Alliance A031701 (NCT03609216) [96]	Multicenter, prospective, phase II trial	Standard dose or dose-dense gemcitabine and cisplatin	▪ 271 patients with cT2-T4aN0/xM0 MIBC ▪ Primary tumors contain deleterious alterations in any 1 of 9 pre-selected DDR genes (ERCC2, ERCC5, BRCA1, BRCA2, RECQL4, RAD51C, ATM, ATR, and FANCC) and who exhibit <T1 response on clinical restaging are eligible for organ-sparing management. ▪ Primary endpoint: 3-year event-free survival in DDR-altered pts who undergo bladder sparing, defined as the proportion of patients without BCG-unresponsive non-muscle-invasive recurrences, any >T2 recurrences, or any metastatic recurrences.
HCRN GU16-257 (NCT03558087) [97]	Prospective	4 cycles of gemcitabine, cisplatin, plus nivolumab	▪ 76 patients with cT2-T4aN0M0 MIBC ▪ Patients achieving a cCR (normal cytology, imaging, and cT0/Ta) were eligible to proceed without cystectomy and receive nivolumab q2 weeks x 8 followed by surveillance. ▪ Primary endpoint: (1) cCR rate (2) ability of cCR to predict 2-year metastasis-free survival (MFS) ▪ Interim results: Local recurrence has occurred in 8/31 cCR patients and 6 underwent RC (pT0N0 = 1, pTaN0 = 1, pTisN0 = 1, pT2N0 = 2, pT4N1 = 1). TMB ≥ 10 mut/Mb (*p* = 0.02) or mutant ERCC2 (*p* = 0.02) were associated with cCR or pT0.
